# Increasing sensitivity to DNA damage is a potential driver for human ovarian cancer

**DOI:** 10.18632/oncotarget.10436

**Published:** 2016-07-06

**Authors:** Yimei Jin, Xin Xu, Xuemeng Wang, Henry Kuang, Michael Osterman, Shi Feng, Deqiang Han, Yu Wu, Mo Li, Hongyan Guo

**Affiliations:** ^1^ Department of Obstetrics and Gynecology, Peking University Third Hospital, Beijing, 100191, P.R. China; ^2^ Department of Molecular and Medical pharmacology, University of California, Los Angeles, 90095, USA; ^3^ Medical School and University of Michigan, Ann Arbor, Michigan, 48109, USA; ^4^ School of Public Health, University of Michigan, Ann Arbor, Michigan, 48109, USA; ^5^ Education Department, Peking University Third Hospital, Beijing, 100191, P.R. China; ^6^ Department of Biochemistry and Molecular Biology, State Key Laboratory of Medical Molecular Biology, Institute of Basic Medical Sciences Chinese Academy of Medical Sciences, Peking Union Medical College, Beijing, 100005, China

**Keywords:** ovarian cancer, DNA damage, DNA repair, genomic instability

## Abstract

Ovarian cancer is one of the most common cancers among women, accounting for more deaths than any other gynecological diseases. However, the survival rate for ovarian cancer has not essentially improved over the past thirty years. Thus, to understand the molecular mechanism of ovarian tumorigenesis is important for optimizing the early diagnosis and treating this disease. In this study, we observed obvious DNA lesions, especially DNA double strand breaks (DSBs) accompanying cell cycle checkpoint activation, in the human epithelial ovarian cancer samples, which could be due to the impaired DNA response machinery. Following this line, we found that these DNA damage response-deficient primary cancer cells were hypersensitive to DNA damage and lost their ability to repair the DNA breaks, leading to genomic instability. Of note, three key DNA damage response factors, RNF8, Ku70, and FEN1 exhibited dramatically decreased expression level, implying the dysfunctional DNA repair pathways. Re-expression of wild type RNF8, Ku70, or FEN1 in these cells restored the DNA lesions and also partially rescued the cells from death. Our current study therefore proposes that accumulated DNA lesions might be a potential driver of ovarian cancer and the impaired DNA damage responders could be the targets for clinical treatment.

## INTRODUCTION

Ovarian cancer is the major cause of death in the gynecological cancers and among the most common malignancies for women. The estimated annual incidence of ovarian cancer is more than 200,000 in the world. And this number is much increased in developing countries [1]. Approximately seventy percent of ovarian cancer patients are diagnosed at their advanced stage with wide metastasis within the peritoneal cavity, and only a small number of them can be expected to survive five years [2, 3]. Due to the absence of specific signs, ovarian cancer shares many symptoms with common gastrointestinal, genitourinary and gynecological conditions, that blocks the early diagnosis of this disease [4]. But if diagnosed at the early stage, around ninety percent of patients can be cured by the current clinical approaches [5]. Thus, developing effective screening could not only advance the early diagnosis, but also provide potential targets for the therapy.

The most lethal ovarian cancer is epithelial ovarian cancer (EOC), which accounts for ninety percent of all ovarian cancers [6]. Scientists have given much effort towards deciphering the etiologic factors of ovarian cancer such as early age at menarche, delayed menopause, genetic alteration, use of estrogen and hormone replacement therapy [7–10]. However, the survival rate for this disease has not essentially changed during the last thirty years. Previous studies have revealed various biomarkers for the diagnosis of EOC which shows clinical significance [11–14]. But the mechanical information has not yet been translated into clinical treatment. Thus, research focusing on the molecular changes underlying ovarian cancer needs to be conducted to identify crucial factors of signaling ways responsible for the initiation and progression of this gynecological malignancy.

Accumulative evidence has demonstrated that DNA damage in our body could be the culprit for tumorigenesis [15–17]. It has been known that every cell encounters up to 10^6^ DNA lesions per day, which can be induced by exogenous physical agents, spontaneous chemical reactions, and products of endogenous metabolism [18]. Most of the damages could be sensed and fixed timely by diverse pathways of DNA repair such as base excision repair (BER), homologous recombination (HR), as well as non-homologous end joining (NHEJ) in the DNA damage response system [19–21]. If these lesions are not repaired, however, they could be a potential risk for the local tissue. Among the DNA damages, DNA double strand breaks (DSBs) are the most deleterious lesion since no intact complementary strand is left as a template for the repair. Failure of DSBs repair results in genomic instability, and consequently tumorigenesis [22, 23]. Thus, the well-running machinery of the DNA damage response system is the guard against genomic insult and tumorigenesis.

In this study, we observed obvious DNA lesions especially DSBs accompanying cell cycle checkpoint activation, in the human epithelial ovarian cancer samples which could be resulted from the impaired DNA response machinery. Given these results, we found that these DNA damage response-deficient primary cancer cells were hypersensitive to DNA damage and had little ability to repair the DNA breaks. Importantly, three key DNA damage responders, RNF8, Ku70, and FEN1 showed dramatically decreased expression level, implying the dysfunction of DNA repair pathways. Re-expressing wild type RNF8, Ku70, or FEN1 in these primary cancer cells restored the DNA breaks and partially rescued the cells from DNA damage-induced death. Here, our work proposes that accumulated DNA lesions may be an original source of ovarian cancer and the impaired DNA damage responders could be screened as markers for early diagnosis and therapy targets.

## RESULTS AND DISCUSSION

### Massive DNA damages are present in human ovarian cancer tissue

**Figure 1:**
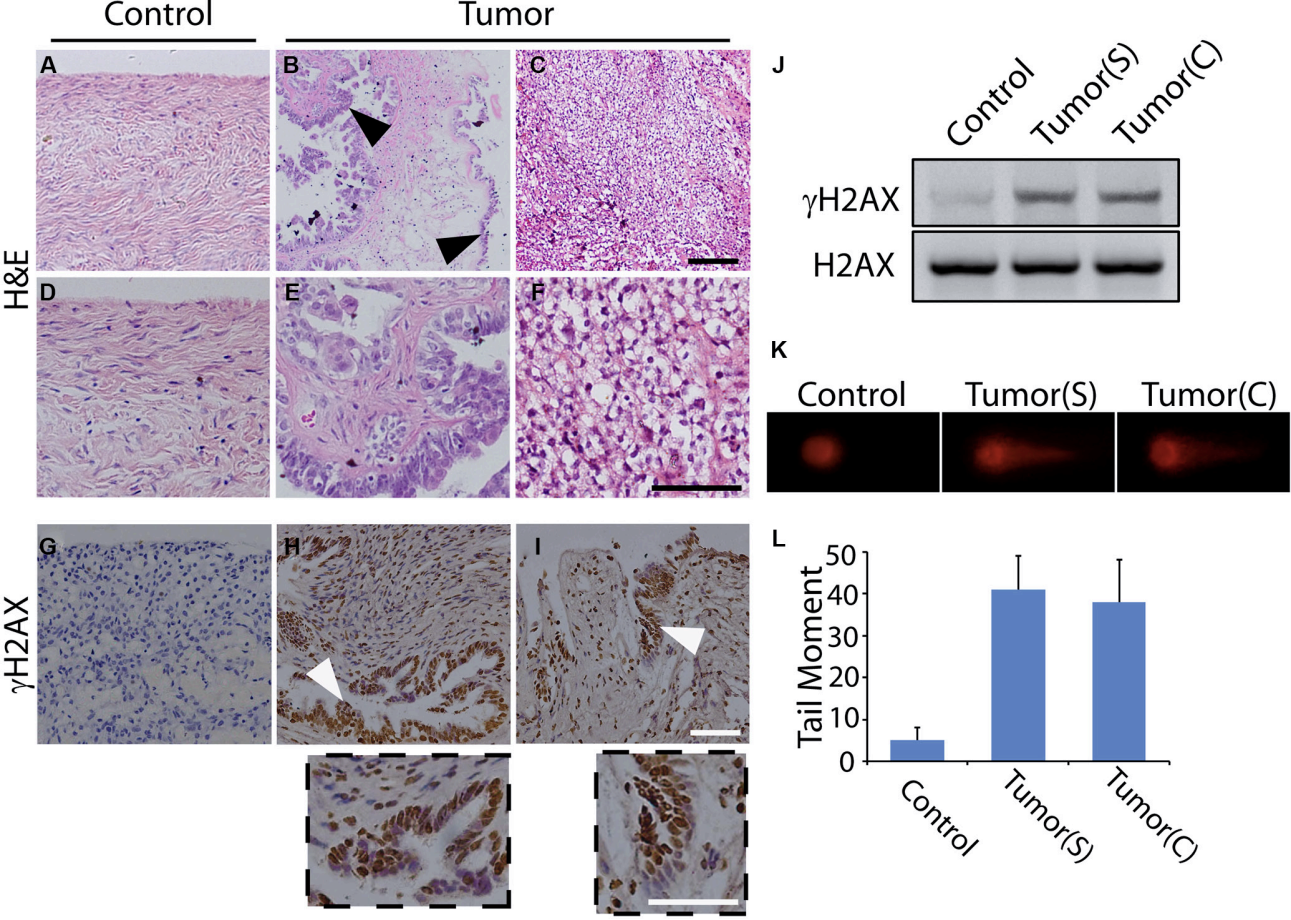
DNA damage occurs in human ovarian cancer (**A–C**) H&E staining shows the histomorphology of normal ovarian tissue (A), serous ovarian cancer tissue (B), and clear cell ovarian cancer tissue (C). (**D–F**) Exhibited higher magnification for A-C, respectively. (**G–I**) Immunohistochemistry of γH2AX in the normal (G), serous ovarian cancer (H), and clear cell ovarian cancer tissues (I). The below insets showed the higher magnification for H and I, respectively. Representative carcinomatous regions were indicated by arrowheads. Scale bars, 100 μm. (**J**) γH2AX was detected by Western Blot in para-tumor control, serous ovarian cancer, and clear cell ovarian cancer tissues. H2AX was used as the loading control. (**K–L**) DSBs in primary cells from para-tumor and ovarian cancer tissues are examined by neutral comet assay and measured by tail moment. Tumor(S) indicates the serous ovarian cancer tissue, and Tumor(C) indicates the clear cell ovarian cancer tissue.

To study the tumorigenesis of ovarian cancer, we harvested the fresh ovarian tissues from our hospital. Ten ovarian cancer samples were randomly selected from 38 cases of ovarian cancer patients. These cases included six females diagnosed with serous ovarian cancer (T1- T6) and four females with clear cell ovarian cancer (T7-T10) (Table 1). Their average age was 51 years and all the patients showed representative clinical symptoms, such as persistent pelvic/abdominal pain and increased abdominal size. Eight patients had lymph node metastasis, and four had peritoneal metastasis. Representative morphologies of normal epithelium were shown in the para-tumor controls by hematoxylin and eosin (H&E) staining in Figure 1A. For the samples from the serous ovarian cancer patients, papillary formations were complex and multilayered with the nests or undifferentiated sheets of malignant cells invading the axial fibrous tissue. Some areas of the tissues exhibited deep blue stained hyperchromatic nuclei enlarged with pleomorphic change (Figure 1B). In the samples from clear cell ovarian cancer patients, cells with clear cytoplasm that contained glycogen invaded the axial fibrous tissue (Figure 1C). Figure 1D–1F exhibited the enlarged pictures for Figure 1A–1C. Moreover, the primary cells from the tumor and para-tumor control were harvested, and their growth rate was examined by MTT assay. As expected, most of the tumor cells (6 out of 8 texted samples) gave higher proliferation than the controls (Supplementary Figure S1A).

**Table 1:** Characteristics of the patients and tumors

NO.	Age	Tumor size (cm)	Grade	Lymph node metastasis	Peritoneal metastasis
T1	45	5.0 ×4.2	serous/low-IV	+	+
T2	41	7.8 ×5.5	serous/high-IIIb	+	+
T3	53	4.7 ×3.9	serous/high-IIb	+	−
T4	63	5.2 ×4.0	serous/low-III	+	−
T5	48	4.8 ×4.8	serous/low-II	−	−
T6	61	3.7 ×4.2	serous/high-IIb	+	+
T7	50	5.2 ×4.8	clear cell/IIb	−	−
T8	49	4.6 ×4.3	clear cell/IIIa	+	−
T9	53	5.8 ×4.3	clear cell/IIb	+	+
T10	47	5.2 ×3.9	clear cell/IIb	+	−

To examine the relationship between DNA damage and ovarian tumorigenesis, we first detected the occurrence of DNA damages in these ovarian samples. It is well known that H2AX, the variant of canonical histone H2A, plays a critical role in signal spreading of the DNA damage, and phosphorylated H2AX (γH2AX) is required for the stabilization of various DNA damage response factors at DNA damage sites [24, 25]. Thus, γH2AX is usually employed for the surrogate marker of DSBs presence [26–28]. Immunohistochemistry of these samples was performed with the γH2AX antibody. Of note, obviously positive signals were found in all of the ovarian cancer samples, while little positive staining was detected in the para-tumor controls (Figure 1G–1I). These results were verified by Western Blot of γH2AX (Figure 1J). To further confirm the presence of DNA damages, we isolated the primary cells from the fresh samples followed by the neutral comet assay to examine DSBs. As seen in Figure 1K and 1L, cells with long comet tails were detected among the cells from the tumor samples rather than from the controls, indicating that DNA damages occurred in these ovarian cancer tissues. Besides, DNA single strand breaks (SSBs) were tested in these samples by staining 8-oxoguanine DNA glycosylase (OGG1), the core member of base-excision repair (BER) for SSBs repair [21]. As seen in Supplementary Figure S1B, SSBs occurred in some of the tested samples (5/9) with different intensities of OGG1 staining in the nuclei. And these SSBs were accompanied with the activation of ATR (Supplementary Figure S1C). Thus, these data reveal a close relationship between DNA damage and ovarian tumorigenesis.

### DNA damage response and cell cycle checkpoint are activated in human ovarian cancer tissue

**Figure 2:**
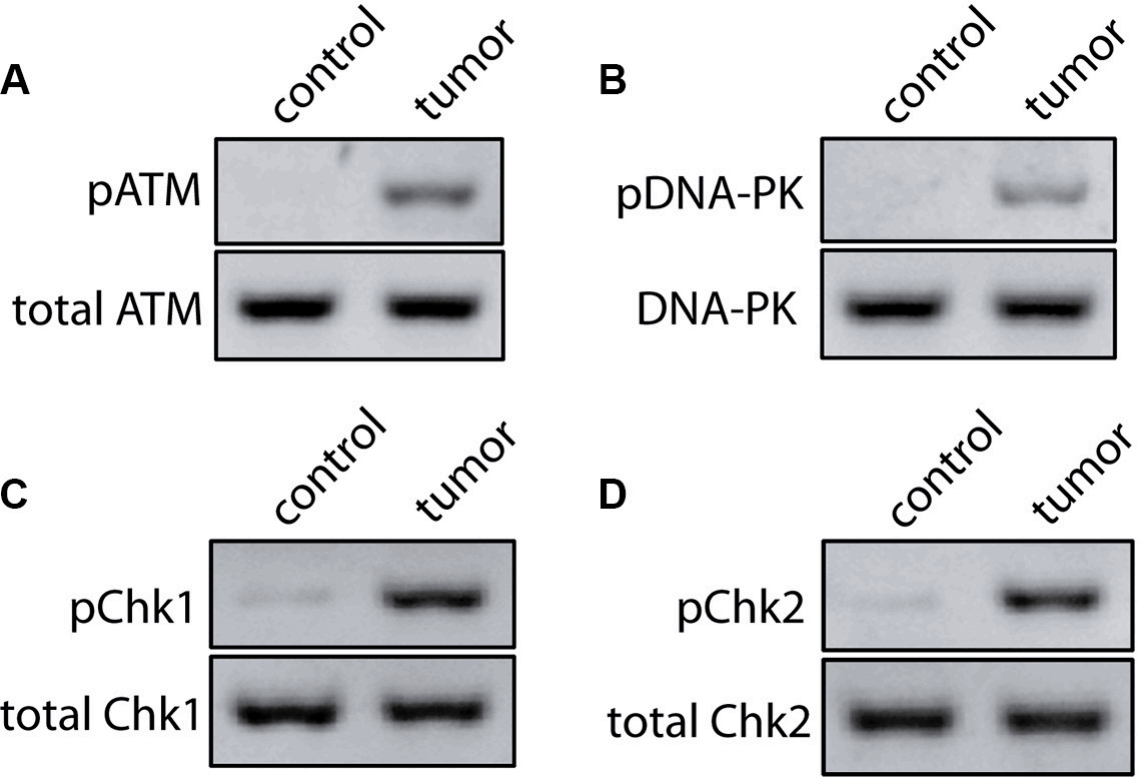
Activated DNA damage response and cell cycle checkpoint in human ovarian cancer (**A, B**) Phosphorylation status of ATM and DNA-PK was tested by Western Blot in para-tumor and tumor ovarian tissues. Total ATM and DNA-PK were used for the loading controls, respectively. (**C, D**) Phosphorylation status of Chk1 and Chk2 was tested by Western Blot in para-tumor and tumor ovarian tissues. Total Chk1 and Chk2 were used for the loading controls, respectively.

To encounter DNA damages, cells evolve DNA damage response system to protect the integrity of the genome by activating cell cycle checkpoint, initiate the DNA repair program, or promote the cells to apoptosis [15, 29, 30]. In the DNA damage response system, ataxia-telangiectasia mutated (ATM) and DNA-dependent protein kinase (DNA-PK) are known to be the earliest sensors of DNA lesions and are rapidly activated for the launch of DNA damage response [25, 31, 32]. Thus, we investigated whether the ATM and DNA-PK were activated in these samples. Importantly, all cancer samples showed the active ATM (phosphorylated ATM) and active DNA- PK (phosphorylated DNA-PK catalytic subunit, pDNA- PKcs), even though the activated strengths among these tissues were not strictly equal. However, little active ATM and DNA-PK were detected in the para-tumor controls (Figure 2A and 2B). Since higher level of pATM may trigger apoptosis, we also examined the apoptosis signal in these tissues. As expected, some regions of four samples (4 out of 10) showed obvious cleaved caspase 3 staining (Supplementary Figure S2). It is not surprising that some regions of tested samples undergo apoptosis. ATM is one of the earliest sensors upon DNA damage. Active ATM triggers cell cycle activation which allows DNA repair machinery to have enough time to repair DNA lesions. If the DNA damage is too heavy to be repaired, cells launch apoptosis signals [33, 34]. So the active ATM could result in different fates for cells, including at least survival and apoptosis. It is well known that ATM and DNA-PK are the key sensors of DNA double-strand breaks (DSBs) in HR and NHEJ, respectively [25, 31], these results therefore indicate that DSBs are closely related with ovarian cancer. Compared with other DNA lesions, DSBs are more lethal to our body because both of the DNA strands are impaired and thus no intact template is left for replication during the repair, which leads to chromosomal breakage and rearrangement, and finally genomic instability [22, 35]. Also, an increasing number of studies show evidence that deficiencies in DSBs repair are fundamental to the etiology of most, if not all, human cancers [23, 36, 37]. We thus assume that DNA damage may be an essentially potential driver of human ovarian cancer.

It has been shown that the active ATM and DNA-PK phosphorylate the downstream substrates checkpoint kinase 1 (Chk1) and checkpoint kinase 2 (Chk2) [31, 38], which inactivate the CDC25 family of dual-specificity phosphatases and stop the cell cycle [38–40]. Thus, we tested the active status of both Chk1 and Chk2 in the ovarian tissues by using their phosphorylation antibodies. Little active Chk1 or Chk2 was found in the control samples. However, these kinases were obviously activated in the tumors (Figure 2C and 2D), indicating that in the tumor cells, due to the persistent DNA damage, the cell cycle checkpoint was over-activated. In particular, the G2/M checkpoint is only a quite transient cell cycle arrest at the G2/M boundary which occurs immediately following DNA damage [41]. Thus, the persistent arresting at the G2/M transition induced by the over-activated Chk may result in the mitotic exit and genomic instability [25, 42–44], and this could be the driver of ovarian tumorigenesis. Further study aiming for the accurate cell cycle investigation of the primary cancer cell *in situ* derived from the tumor tissue would provide more insight on the precision medicine for ovarian tumorigenesis.

### Primary cells from human ovarian cancer tissue are hypersensitive to DNA damage

**Figure 3:**
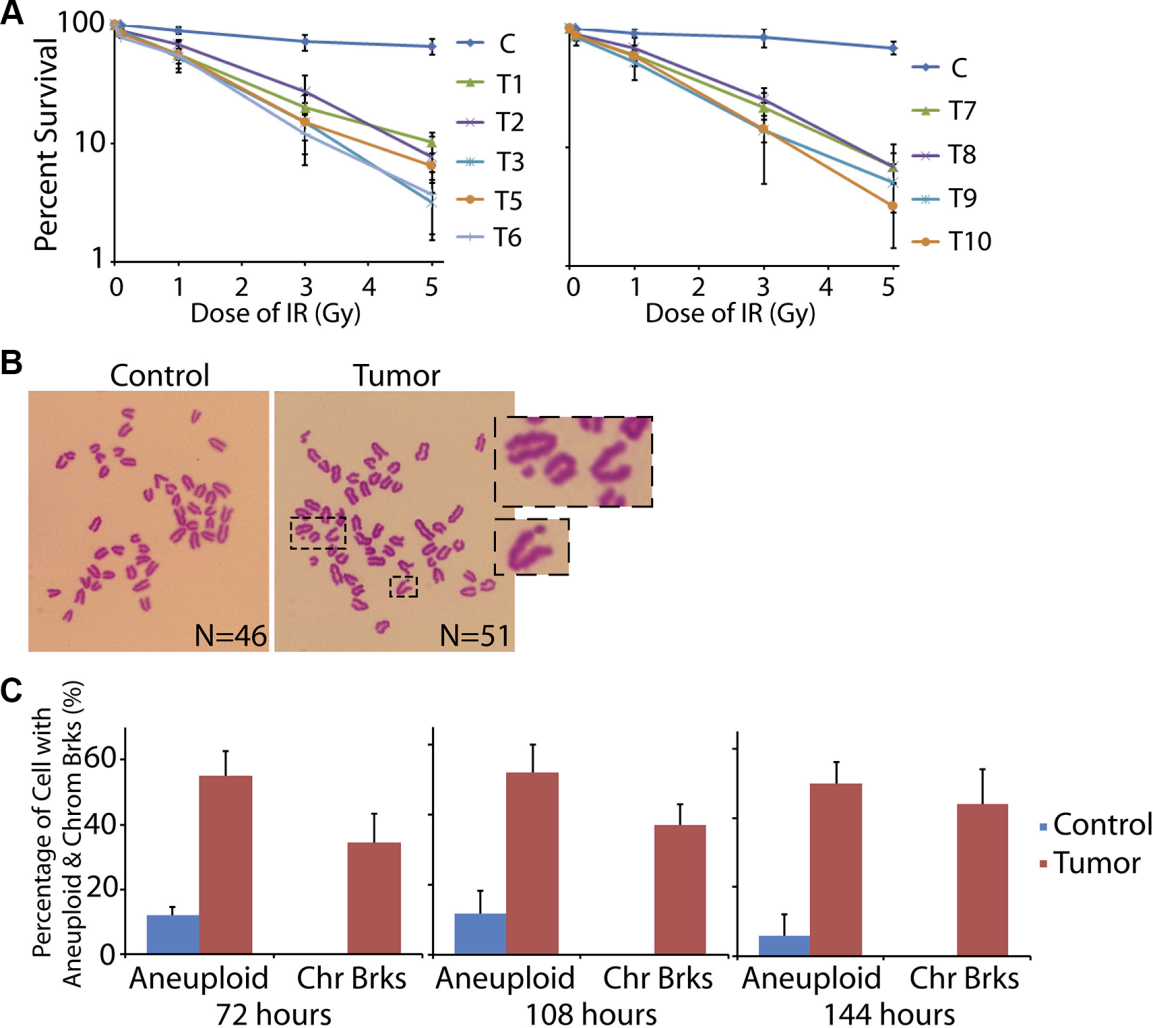
Cells from ovarian cancer tissue are hypersensitive to DNA damage (**A**) Primary cells from control and cancer tissues were treated by low dose of IR followed by the living cell counting. More than 89% of cancer cells died within 7 days, significantly higher than that in control group. The error bars represent the standard deviation. (**B, C**) Primary cells from control and cancer tissues were treated by low dose of IR followed by mitotic spreads. Most of the cancer cells showed aneuploidy and broken chromosomes after 3, 4.5, and 6 days recovery from the IR.

Since massive DNA damages were found in the ovarian cancer tissue, we assumed that the DNA repair system in these cancerous cells was impaired. Thus, we harvested the primary cells from the fresh ovarian specimens and examined the DNA repair ability. The primary cells were cultured *in vitro* and treated with or without ionizing radiation (IR). Notably, 89.8%–96.8% of the tumor cells went to death within one week after the IR exposure (for T04 tumor sample, we did not have enough primary cells for the assay, and thus omitted this group in this part), while most cells from the controls survived from the treatment (Figure 3A).

If DSBs are accumulated in the genome without timely repair, they could result in chromosome breaks. Thus, after 72 hours of the recovery from IR exposure, we tested the chromosome status of the primary cells by mitotic spreads. As expected, most of the cells in control tissue exhibited normal chromosome pattern with 46 chromosomes. However, cells from the tumor sample showed broken chromosomes, accompanied with obvious aneuploidy (Figure 3B and 3C). When we prolonged the recovery time to 108 and 144 hours, the chromosome breaks and aneuploidy could be still detected (Figure 3C). These data suggested that the DNA repair ability in these cancer cells was impaired, and thus the IR-induced DSBs were not fixed.

### The expression of DNA damage response factors are disturbed in human ovarian cancer tissue

**Figure 4:**
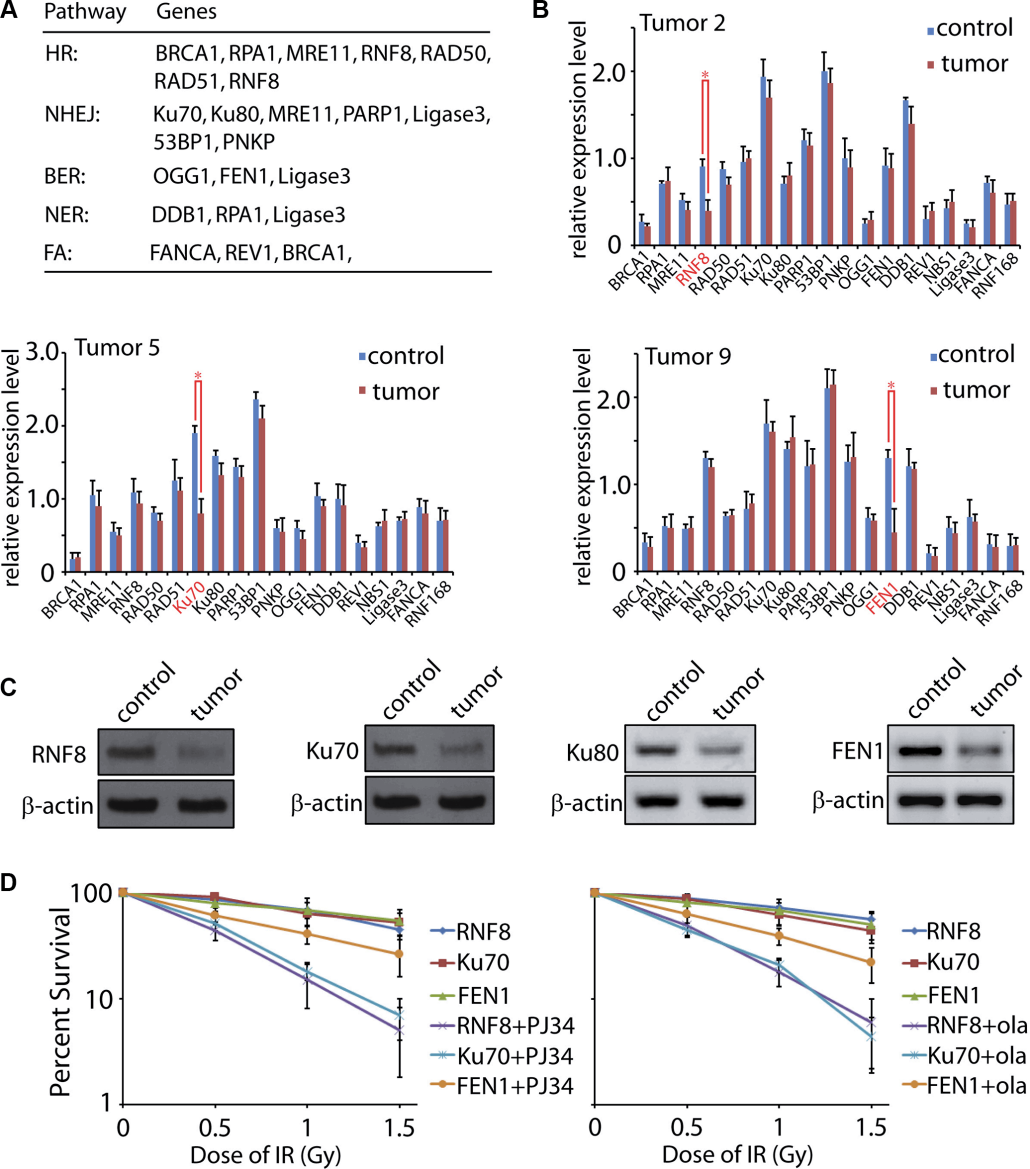
Expression level of DNA damage response factors in human ovarian cancer (**A**) A general pattern of DNA damage response factors in HR, NHEJ, BER, NER, and FA pathways. The included members function mainly, but not limited to, in the indicated pathways. (**B**) Expression level of the nineteen DNA damage response factors in control and tumor tissues was tested by qRT-PCR. Representative data were summarized from T2, T5, and T9 samples. (**C**) Expression levels of RNF8, Ku70, Ku80, and FEN1 were tested by Western Blot with the indicated antibodies. β-actin was used as the loading control. (**D**) Primary cells from RNF8, Ku70, and FEN1 deficient tumor tissues were treated by low dose of IR in the presence of mock or PARP inhibitor (PJ34 or olaparib) followed by the living cell counting. The error bars represent the standard deviation.

Since DNA repair system was deficient in the ovarian cancer tissues, we hypothesized that factor(s) for DNA damage response may be impaired. The expressions of key factors of DNA damage response were tested by quantitative reverse transcription PCR (qRT-PCR). Here we selected nineteen candidates of the repair factors including BRCA1, RPA1, RNF8, RAD50, RAD51, MRE11, Ku70, Ku80, PARP1, 53BP1, PNKP, OGG1, FEN1, DDB1, REV1, NBS1, Ligase3, FANCA, and RNF168 (Figure 4A). These factors are proposed to function mainly, but not limited to, in HR, NHEJ, BER, nucleotide excision repair (NER), and fanconi anemia (FA) [17, 19, 20, 23, 33, 45–48]. The primers used for the quantification were listed in Supplementary Table S1. Notably, we found that the expressions of RNF8, Ku70, and FEN1 were significantly decreased in three tumor samples (T2, T5, and T9), respectively (Figure 4B).

To confirm the qRT-PCR results, we examined the protein levels of these three candidates in the tumor samples. As expected, the T2, T5 and T9 tissues showed lower protein level of RNF8, Ku70, and FEN1, respectively, correlated to their mRNA expression levels (Figure 4C). Since Ku70 and Ku80 form a stable heterodimeric complex *in vivo* [49, 50], it is expected that Ku80 would be reduced if Ku70 is down-regulated. No significant difference of Ku80 mRNA was found between the para-tumor and tumor tissues. However, the protein level exhibited a decrease, suggesting that loss of Ku70 led to an unstable status of the functional partner Ku80. It is no surprising that not all the tumor samples showed the dramatic expression change of the tested DNA repair factors. In the DNA damage response system, besides the nineteen candidates we selected, numerous other factors also participate in the repair progress such as Ligase4, CtIP, PALB2, XLF, PCNA, APTX, and other members in the RAD family [34, 51, 52]. Moreover, many pathways are regulated by post-transcriptional modification such as phosphorylation, acetylation, ubiquitination, as well as ADP-ribosylation [26, 53, 54]. Thus, the abnormal expression of RNF8, Ku70, and FEN1 may only show a fraction of impaired DNA damage factors. More detailed investigation focusing on the post-transcriptional modification, mutations screening, and structure biology will provide mechanistic insights on the dysregulation of DNA damage response. Although the expression deficiency of DNA repair factors is different among tumors, the resulting outcome is similar, that is, the DNA repair ability in each sample was impaired. Thus, these unrepaired DNA lesions could eventually lead to genomic instability [15, 22, 29]. Also, the most deleterious DNA damage, DSBs, could be generated directly and indirectly. For instance, Ku70 deficiency results in the failure of DSBs repair [52], while FEN1 dysfunction leads to SSBs [55]. Accumulated SSB lesions will be duplicated during DNA replication, which converts into DSBs. Alternatively, if two SSBs during BER in the complement strands locate close to each other, DSBs may also occur naturally [17].

Recently, PARP inhibitor is emerging as the promising chemotherapy drug against breast and ovarian cancers [56–59], and cells with repair deficiency for DSBs are hypersensitive to this compound [60, 61]. Thus, we investigated the chemotherapy effect of PARP inhibitor on the RNF8, Ku70, and FEN1 deficient primary cancer cells. As expected, both RNF8 and Ku70 deficient cancer cells were hypersensitive to PJ34 and olaparib, the two widely used PARP inhibitors [27, 59]. However, the chemotherapy efficiency on FEN1 deficient cells was weaker than those on RNF8 and Ku70 deficient cells (Figure 4D). Previous study reported that cells losing DSBs repair ability are more dependent on PARP for their survival since PARP functions mainly on BER pathway [56]. As FEN1 mainly functions in BER [45, 55], PARP inhibitor could not further hit these FEN1 deficient cells.

### Re-expression of DNA damage response factors decreases DNA damage in ovarian cancer cells

**Figure 5:**
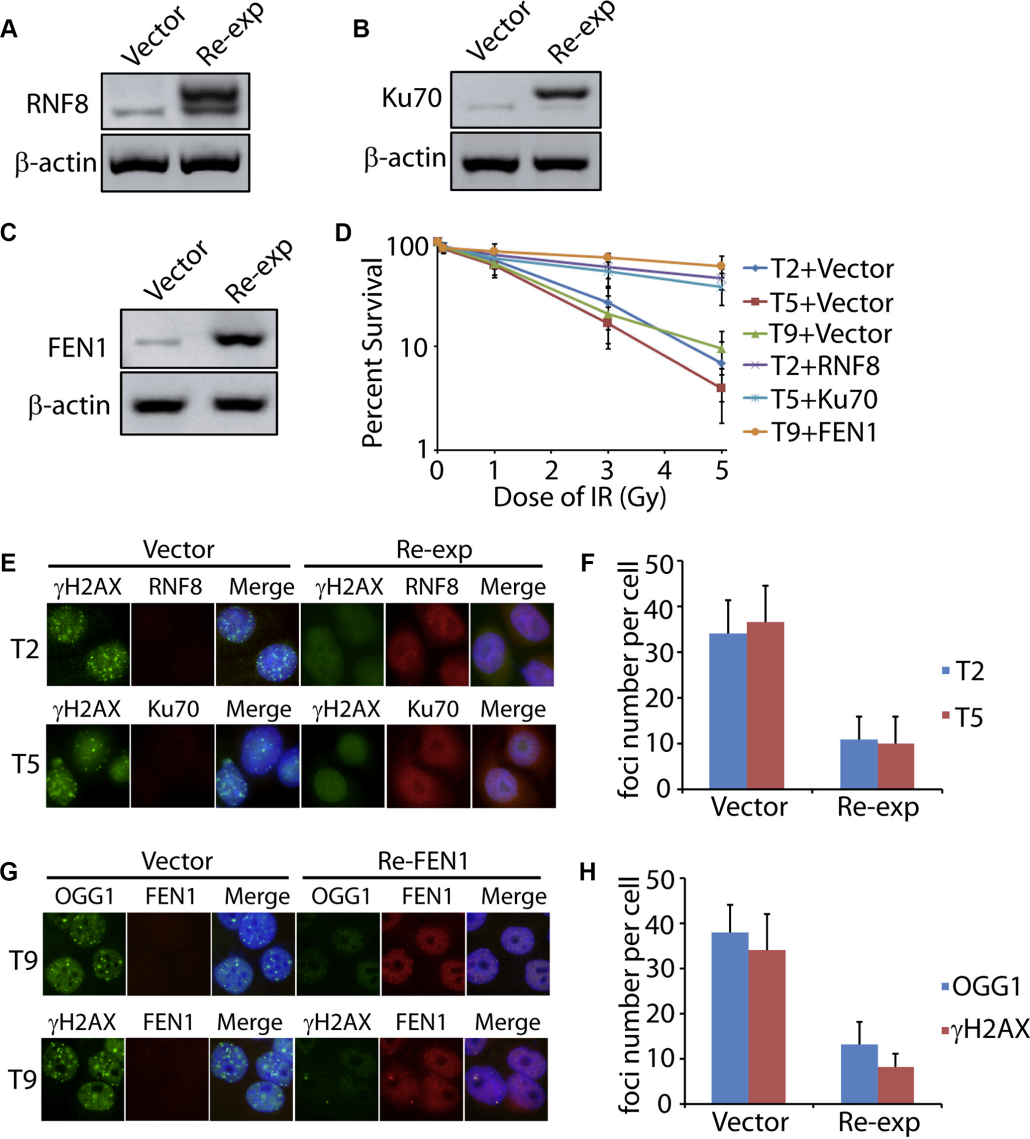
Re-expression of DNA repair factors rescues cancer cells from DNA damage (**A–C**) Wild type RNF8, Ku70, and FEN1 with flag tag were expressed in T2, T5, and T9 primary cells. The re-expressed proteins were detected by anti-RNF8, anti-Ku70, and anti-FEN1 antibodies, respectively. β-actin was used as the loading control. (**D**) Primary cancer cells from T2, T5, and T9 tissues re-expressed with or without RNF8, Ku70, or FEN1 were treated by IR followed by living cell counting. (**E–H**) Primary cancer cells from T2, T5, and T9 tissues re-expressed with or without RNF8, Ku70, or FEN1 were treated by 5 Gy IR followed by immunofluorescence of γH2AX or OGG1. The foci number in each cell was counted. For the re-expressed groups, only cells with positive RNF8, Ku70, or FEN1 staining were recorded. Each subgroup included 50 cells. The error bars represent the standard deviation.

Since RNF8, Ku70, and FEN1 showed the low expression level in the tumor tissues (T2, T5, and T9) with DNA damage, we proposed that the DNA damage might be resulted from these impaired DNA repair machineries. Human wild type RNF8, Ku70, and FEN1 were subcloned into the Flag-tagged plasmid. Primary cells from the tumor samples were harvested and cultured, followed by the re-expression of the wild type RNF8, Ku70, and FEN1, respectively (Figure 5A–5C). Cells were then treated with IR and cell death was recorded, comparing between the mock and re-expressed groups. As seen in Figure 5D, only 6.9% of the RNF8 deficient cells (T2), 3.9% of the Ku70 deficient cells (T5), and 9.5% of the FEN1 deficient cells survived seven days after the IR assault. Interestingly, 44.6%, 37.1%, and 59% cells were restored by the wild type RNF8, Ku70, and FEN1, respectively. We further tested the DNA damage by immunofluorescence of γH2AX or OGG1 in the cells. As expected, persistent γH2AX foci were observed in the cells from T2, T5, and T9 tumor tissues, and OOG1 foci were observed in the cells from T9 sample. However, both the foci number and intensity were decreased in the wild type RNF8, Ku70, and FEN1 re-expressed cells (Figure 5E–5H), indicating that regain of normal DNA repair machinery could, at least partially, rescue the IR-induced DNA damage.

Remarkably, an increasing body of evidence has shown the close relationship between DNA damage and tumorigenesis [16, 62, 63]. Recent study found that down-regulation of RAD51 by microRNA augmented the chemotherapy response in the treatment of serous ovarian cancer [64]. Similarly, decreasing the expression of RNF8 by microRNA resulted in the impaired DNA repair and induced chromosomal instability in ovarian cancer cells [65]. Also, the expression of numerous DNA repair genes was evidenced to be misregulated in ovarian carcinoma caused by the mutation of CDK12, and the CDK12 mutation is one of the major recurrent somatic mutations in high-grade serous ovarian carcinoma [66]. Each cell in our body encounters nearly 10^6^ DNA damages per day and these genomic assaults come from the endogenous metabolism and exogenous physical agents [17, 18]. Most of these lesions can be sensed and fixed by DNA damage response system. Thus, in this DNA damage response network, dysfunction of one or multiple key factor(s) such as BRCA1, RNF8, Ku70 or NBS1, could cause the DNA response failure and genomic instability. The current medicine strategy against ovarian cancer emphasizes targeting the dominant factor(s) during the clinical trials. One of the efficient approaches is developed by trying to identify the germline and sporadic key mutations in the primary site of the tumors [67–69]. The study here not only reveals the DNA damage as a potential driver of ovarian tumorigenesis, but also provides possible gene targets for clinical treatment.

## MATERIALS AND METHODS

### Chemicals and antibodies

All chemicals and media were purchased from Sigma Chemical Company (St. Louis, MO) except for those specifically mentioned. Anti- γH2AX, pATM, ATM, pChk1, Chk1, pChk2, Chk2 antibodies were purchased from Cell Signaling. Anti- pDNA-PKcs, RNF8, Ku70, and FEN1 antibodies were purchased from Abcam.

### Samples

Ovarian tissues were collected from patients at Peking University Third Hospital in China after informed consent was obtained from all the patients. Briefly, tissues were obtained from the operating room from the consenting donors. After PBS wash, each tissue was separated into three parts which were fixed in 10% Buffered Formalin for at least 16 hours, frozen at −80°C, and frozen in liquid nitrogen, respectively. All the samples used in this study were harvested after obtaining approval from the ethics committees at Peking University Third Hospital and Peking University.

### Hematoxylin and eosin (H&E) staining

Tissues from both experimental and control samples were fixed overnight in 10% neutral buffered formalin, embedded in paraffin, and sectioned. Embedding and sectioning were performed by the Immunohistochemistry Core at Peking University Third Hospital. Sections were then subjected to hematoxylin and eosin staining.

### Western blot

Protein samples from the tissues were extracted by using a total protein extraction kit (Millipore, #2140). The proteins were separated by SDS-PAGE and then electrically transferred to polyvinylidene fluoride membranes. Following transfer, the membranes were blocked in TBST (TBS containing 0.1% Tween 20) containing 5% skimmed milk for 2 hours, followed by incubation overnight at 4°C with the indicated primary antibodies, respectively. After washing in TBST, the membranes were incubated for 1 hour at room temperature with 1:1000 horseradish peroxidase (HRP)-conjugated IgG. To detect total ATM, DNA-PK, Chk1 or Chk2, the membranes were washed in the stripping buffer (100 mM β-mercaptoethanol, 20% SDS, and 62.5 mM Tris, pH 6.7) for 30 minutes at 55°C, and then subjected to another round of incubation. Finally, the membranes were detected by the enhanced hemiluminescence detection system (Amersham, Piscataway, NJ).

### Isolation and culture of primary ovarian cancer cells

The isolation and culture of primary ovarian cancer cells was performed according to the previous report [70]. Briefly, the fresh clinical specimens of ovarian cancer are collected after surgery and cut into small pieces constituting the cell slurry. The minced tissues were then exposed to DMEM with 2.4 U/ml dispase II for 30 mins at 37°C. After that, the cell slurry was transferred onto a cell strainer to separate EOC cells from any undissociated tissue. The recovered cell suspension was placed at 5% CO_2_ and 37°C. By day 6–7, EOC cells formed swirl-like shapes and started to spread to the plastic to form larger multicellular aggregates leading to the typical cobblestone morphology of EOC cells. By day 14, typical EOC cells were formed and were ready for downstream experiments.

### MTT assay

The cell proliferation rate was measured by 3-(4,5-dimethylthiazol-2-yl)-2,5-diphenyltrazolium bromide (MTT) assay. In brief, 2 × 10^3^ cells were seeded on each well of 96-well culture plates and cultured for 48, 96, and 120 hours, respectively. After the indicated time points of the culture, 10 μl of MTT reagent was added to the cells, followed by another 4 hours of incubation at 37°C. DMSO was added to dissolve the formazan product for 10 minutes at room temperature. Finally, the absorbance was measured at 492 nm using a microplate reader (Bioteck Powerwave^™^, USA).

### Immunofluorescence microscopy

Immunofluorescence was performed as described previously [71]. In brief, tissues were fixed in 4% paraformaldehyde in PBS (pH 7.4) for at least 3 hours at room temperature. After being permeabilized with 0.5% Triton X-100 at room temperature for 30 minutes, tissues were blocked in 1% BSA-supplemented PBS for 1 hour and incubated overnight at 4°C with the indicated antibodies, respectively. After washing 3 times in PBS containing 0.1% Tween 20 and 0.01% Triton X-100 for 5 minutes each, the tissues were labeled with 1:500 FITC-conjugated IgG or Rho-conjugated IgG for 1 hour at room temperature. After washing in PBS containing 0.1% Tween 20 and 0.01% Triton X-100, the tissues were co- stained with Hoechst 33258 (10 mg/ml in PBS). Finally, the tissues were mounted on glass slides and examined with a fluorescent microscope (Olympus, Japan).

### Ionizing radiation treatment and living cell counting

For the ionizing radiation, cells were irradiated with a 137Cs source at the indicated dose. For living cell counting, five hundred cells were seeded into 6-well plates and cultured for one week. After the culture, the viable cells were harvested, followed by cell counting.

### Comet assay

Single-cell gel electrophoretic comet assay was performed under neutral condition to detect DSBs. Cells were collected and rinsed twice with ice-cold PBS; 2 × 10^4^/ml cells were combined with 1% LMAgarose at 40°C at the ratio of 1:3 (v/v) and immediately pipetted onto slides. For cellular lysis, the slides were immersed in the neutral lysis solution (2% sarkosyl, 0.5 M Na_2_EDTA, 0.5 mg/ml proteinase K in pH 8.0) overnight at 37°C in dark, followed by washing in the rinse buffer (90 mM Tris buffer, 90 mM boric acid, 2 mM Na_2_EDTA in pH 8.5) for 30 minutes with two repeats. The slides were then subjected to electrophoresis at 20 V (0.6 V/cm) for 25 minutes and stained in 2.5 μg/ml propidium iodide for 20 minutes. All images were taken with a fluorescence microscope and analyzed by Comet Assay IV software.

### Statistical analysis

All the experiments were performed at least three times. Results were analyzed using unpaired two-tailed Student's *T*-test and data were expressed as mean ± s.d. *P* values less than 0.05 were considered statistically significant.

## SUPPLEMENTARY MATERIALS FIGURES AND TABLES



## References

[1] Rauh-Hain JA, Krivak TC, Del Carmen MG, Olawaiye AB (2011). Ovarian cancer screening and early detection in the general population. Rev Obstet Gynecol.

[2] Cho KR, Shih Ie M (2009). Ovarian cancer. Annu Rev Pathol.

[3] Jemal A, Siegel R, Ward E, Hao Y, Xu J, Thun MJ (2009). Cancer statistics, 2009. CA Cancer J Clin.

[4] Bast RC, Hennessy B, Mills GB (2009). The biology of ovarian cancer: new opportunities for translation. Nat Rev Cancer.

[5] Das PM, Bast RC (2008). Early detection of ovarian cancer. Biomark Med.

[6] Jelovac D, Armstrong DK (2011). Recent progress in the diagnosis and treatment of ovarian cancer. CA Cancer J Clin.

[7] Tung KH, Goodman MT, Wu AH, McDuffie K, Wilkens LR, Kolonel LN, Nomura AM, Terada KY, Carney ME, Sobin LH (2003). Reproductive factors and epithelial ovarian cancer risk by histologic type: a multiethnic case-control study. Am J Epidemiol.

[8] Fasching PA, Gayther S, Pearce L, Schildkraut JM, Goode E, Thiel F, Chenevix-Trench G, Chang-Claude J, Wang-Gohrke S, Ramus S, Pharoah P, Berchuck A (2009). Role of genetic polymorphisms and ovarian cancer susceptibility. Mol Oncol.

[9] Fortner RT, Ose J, Merritt MA, Schock H, Tjonneland A, Hansen L, Overvad K, Dossus L, Clavel-Chapelon F, Baglietto L, Boeing H, Trichopoulou A, Benetou V (2015). Reproductive and hormone-related risk factors for epithelial ovarian cancer by histologic pathways, invasiveness and histologic subtypes: Results from the EPIC cohort. Int J Cancer.

[10] McLemore MR, Miaskowski C, Aouizerat BE, Chen LM, Dodd MJ (2009). Epidemiological and genetic factors associated with ovarian cancer. Cancer Nurs.

[11] Li M, Hong LI, Liao M, Guo G (2015). Expression and clinical significance of focal adhesion kinase and adrenomedullin in epithelial ovarian cancer. Oncol Lett.

[12] Chen C, Ge J, Lu Q, Ping G, Yang C, Fang X (2015). Expression of Lysine-specific demethylase 1 in human epithelial ovarian cancer. J Ovarian Res.

[13] Timms JF, Arslan-Low E, Kabir M, Worthington J, Camuzeaux S, Sinclair J, Szaub J, Afrough B, Podust VN, Fourkala EO, Cubizolles M, Kronenberg F, Fung ET (2014). Discovery of serum biomarkers of ovarian cancer using complementary proteomic profiling strategies. Proteomics Clin Appl.

[14] Lawrenson K, Mhawech-Fauceglia P, Worthington J, Spindler TJ, O'Brien D, Lee JM, Spain G, Sharifian M, Wang G, Darcy KM, Pejovic T, Sowter H, Timms JF (2015). Identification of novel candidate biomarkers of epithelial ovarian cancer by profiling the secretomes of three-dimensional genetic models of ovarian carcinogenesis. Int J Cancer.

[15] Gorgoulis VG, Vassiliou LV, Karakaidos P, Zacharatos P, Kotsinas A, Liloglou T, Venere M, Ditullio RA, Kastrinakis NG, Levy B, Kletsas D, Yoneta A (2005). Activation of the DNA damage checkpoint and genomic instability in human precancerous lesions. Nature.

[16] Halazonetis TD, Gorgoulis VG, Bartek J (2008). An oncogene-induced DNA damage model for cancer development. Science.

[17] Jackson SP, Bartek J (2009). The DNA-damage response in human biology and disease. Nature.

[18] Ciccia A, Elledge SJ (2010). The DNA damage response: making it safe to play with knives. Mol Cell.

[19] Kim JS, Krasieva TB, Kurumizaka H, Chen DJ, Taylor AM, Yokomori K (2005). Independent and sequential recruitment of NHEJ and HR factors to DNA damage sites in mammalian cells. J Cell Biol.

[20] Shrivastav M, De Haro LP, Nickoloff JA (2008). Regulation of DNA double-strand break repair pathway choice. Cell Res.

[21] David SS, O'shea VL, Kundu S (2007). Base-excision repair of oxidative DNA damage. Nature.

[22] van Gent DC, Hoeijmakers JH, Kanaar R (2001). Chromosomal stability and the DNA double-stranded break connection. Nat Rev Genet.

[23] Khanna KK, Jackson SP (2001). DNA double-strand breaks: signaling, repair and the cancer connection. Nat Genet.

[24] Paull TT, Rogakou EP, Yamazaki V, Kirchgessner CU, Gellert M, Bonner WM (2000). A critical role for histone H2AX in recruitment of repair factors to nuclear foci after DNA damage. Curr Biol.

[25] Osterman M, Kathawa D, Liu D, Guo H, Zhang C, Li M, Yu X, Li F (2014). Elevated DNA damage response in pancreatic cancer. Histochem Cell Biol.

[26] Li M, Lu LY, Yang CY, Wang S, Yu X (2013). The FHA and BRCT domains recognize ADP-ribosylation during DNA damage response. Genes Dev.

[27] Li M, Yu X (2013). Function of BRCA1 in the DNA damage response is mediated by ADP-ribosylation. Cancer Cell.

[28] van Attikum H, Gasser SM (2009). Crosstalk between histone modifications during the DNA damage response. Trends Cell Biol.

[29] Nyberg KA, Michelson RJ, Putnam CW, Weinert TA (2002). Toward maintaining the genome: DNA damage and replication checkpoints. Annu Rev Genet.

[30] Roos WP, Kaina B (2006). DNA damage-induced cell death by apoptosis. Trends Mol Med.

[31] Falck J, Coates J, Jackson SP (2005). Conserved modes of recruitment of ATM, ATR and DNA-PKcs to sites of DNA damage. Nature.

[32] Stiff T, O'Driscoll M, Rief N, Iwabuchi K, Lobrich M, Jeggo PA (2004). ATM and DNA-PK function redundantly to phosphorylate H2AX after exposure to ionizing radiation. Cancer Res.

[33] Sengupta S, Harris CC (2005). p53: traffic cop at the crossroads of DNA repair and recombination. Nat Rev Mol Cell Biol.

[34] Su TT (2006). Cellular responses to DNA damage: one signal, multiple choices. Annu Rev Genet.

[35] Hoeijmakers JH (2001). Genome maintenance mechanisms for preventing cancer. Nature.

[36] Kuschel B, Auranen A, McBride S, Novik KL, Antoniou A, Lipscombe JM, Day NE, Easton DF, Ponder BA, Pharoah PD, Dunning A (2002). Variants in DNA double-strand break repair genes and breast cancer susceptibility. Hum Mol Genet.

[37] Vilenchik MM, Knudson AG (2003). Endogenous DNA double-strand breaks: production, fidelity of repair, and induction of cancer. Proc Natl Acad Sci U S A.

[38] Reinhardt HC, Yaffe MB (2009). Kinases that control the cell cycle in response to DNA damage: Chk1, Chk2, and MK2. Curr Opin Cell Biol.

[39] Bartek J, Lukas J (2003). Chk1 and Chk2 kinases in checkpoint control and cancer. Cancer Cell.

[40] Ng CP, Lee HC, Ho CW, Arooz T, Siu WY, Lau A, Poon RY (2004). Differential mode of regulation of the checkpoint kinases CHK1 and CHK2 by their regulatory domains. J Biol Chem.

[41] Lukas J, Lukas C, Bartek J (2004). Mammalian cell cycle checkpoints: signalling pathways and their organization in space and time. DNA Repair (Amst).

[42] Chiu CC, Li CH, Ung MW, Fuh TS, Chen WL, Fang K (2005). Etoposide (VP-16) elicits apoptosis following prolonged G2-M cell arrest in p53-mutated human non-small cell lung cancer cells. Cancer Lett.

[43] Yang L, Besschetnova TY, Brooks CR, Shah JV, Bonventre JV (2010). Epithelial cell cycle arrest in G2/M mediates kidney fibrosis after injury. Nat Med.

[44] Hirose Y, Berger MS, Pieper RO (2001). p53 effects both the duration of G2/M arrest and the fate of temozolomide-treated human glioblastoma cells. Cancer Res.

[45] Krokan HE, Nilsen H, Skorpen F, Otterlei M, Slupphaug G (2000). Base excision repair of DNA in mammalian cells. FEBS Lett.

[46] de Laat WL, Jaspers NG, Hoeijmakers JH (1999). Molecular mechanism of nucleotide excision repair. Genes Dev.

[47] Walden H, Deans AJ (2014). The Fanconi anemia DNA repair pathway: structural and functional insights into a complex disorder. Annu Rev Biophys.

[48] Tomkinson AE, Sallmyr A (2013). Structure and function of the DNA ligases encoded by the mammalian LIG3 gene. Gene.

[49] Walker JR, Corpina RA, Goldberg J (2001). Structure of the Ku heterodimer bound to DNA and its implications for double-strand break repair. Nature.

[50] Spagnolo L, Rivera-Calzada A, Pearl LH, Llorca O (2006). Three-dimensional structure of the human DNA-PKcs/Ku70/Ku80 complex assembled on DNA and its implications for DNA DSB repair. Mol Cell.

[51] Polo SE, Jackson SP (2011). Dynamics of DNA damage response proteins at DNA breaks: a focus on protein modifications. Genes Dev.

[52] Lieber MR (2010). The mechanism of double-strand DNA break repair by the nonhomologous DNA end-joining pathway. Annu Rev Biochem.

[53] Harper JW, Elledge SJ (2007). The DNA damage response: ten years after. Mol Cell.

[54] Ikura T, Tashiro S, Kakino A, Shima H, Jacob N, Amunugama R, Yoder K, Izumi S, Kuraoka I, Tanaka K, Kimura H, Ikura M, Nishikubo S (2007). DNA damage-dependent acetylation and ubiquitination of H2AX enhances chromatin dynamics. Mol Cell Biol.

[55] Prasad R, Dianov GL, Bohr VA, Wilson SH (2000). FEN1 stimulation of DNA polymerase beta mediates an excision step in mammalian long patch base excision repair. J Biol Chem.

[56] Rouleau M, Patel A, Hendzel MJ, Kaufmann SH, Poirier GG (2010). PARP inhibition: PARP1 and beyond. Nat Rev Cancer.

[57] Fong PC, Boss DS, Yap TA, Tutt A, Wu P, Mergui-Roelvink M, Mortimer P, Swaisland H, Lau A, O'Connor MJ, Ashworth A, Carmichael J, Kaye SB (2009). Inhibition of poly(ADP-ribose) polymerase in tumors from BRCA mutation carriers. N Engl J Med.

[58] Sonnenblick A, de Azambuja E, Azim HA, Piccart M (2015). An update on PARP inhibitors–moving to the adjuvant setting. Nat Rev Clin Oncol.

[59] Oza AM, Cibula D, Benzaquen AO, Poole C, Mathijssen RH, Sonke GS, Colombo N, Spacek J, Vuylsteke P, Hirte H, Mahner S, Plante M, Schmalfeldt B (2015). Olaparib combined with chemotherapy for recurrent platinum-sensitive ovarian cancer: a randomised phase 2 trial. Lancet Oncol.

[60] Farmer H, McCabe N, Lord CJ, Tutt AN, Johnson DA, Richardson TB, Santarosa M, Dillon KJ, Hickson I, Knights C, Martin NM, Jackson SP, Smith GC (2005). Targeting the DNA repair defect in BRCA mutant cells as a therapeutic strategy. Nature.

[61] Li M, Yu X (2015). The role of poly(ADP-ribosyl)ation in DNA damage response and cancer chemotherapy. Oncogene.

[62] Bartkova J, Horejsi Z, Koed K, Kramer A, Tort F, Zieger K, Guldberg P, Sehested M, Nesland JM, Lukas C, Orntoft T, Lukas J, Bartek J (2005). DNA damage response as a candidate anti-cancer barrier in early human tumorigenesis. Nature.

[63] Bartek J, Bartkova J, Lukas J (2007). DNA damage signalling guards against activated oncogenes and tumour progression. Oncogene.

[64] Liu G, Yang D, Rupaimoole R, Pecot CV, Sun Y, Mangala LS, Li X, Ji P, Cogdell D, Hu L, Wang Y, Rodriguez-Aguayo C, Lopez-Berestein G (2015). Augmentation of response to chemotherapy by microRNA-506 through regulation of RAD51 in serous ovarian cancers. J Natl Cancer Inst.

[65] Wang Z, Yin H, Zhang Y, Feng Y, Yan Z, Jiang X, Bukhari I, Iqbal F, Cooke HJ, Shi Q (2014). miR-214-mediated downregulation of RNF8 induces chromosomal instability in ovarian cancer cells. Cell Cycle.

[66] Ekumi KM, Paculova H, Lenasi T, Pospichalova V, Bosken CA, Rybarikova J, Bryja V, Geyer M, Blazek D, Barboric M (2015). Ovarian carcinoma CDK12 mutations misregulate expression of DNA repair genes via deficient formation and function of the Cdk12/CycK complex. Nucleic Acids Res.

[67] Hirotsu Y, Nakagomi H, Sakamoto I, Amemiya K, Oyama T, Mochizuki H, Omata M (2015). Multigene panel analysis identified germline mutations of DNA repair genes in breast and ovarian cancer. Mol Genet Genomic Med.

[68] Ramus SJ, Song H, Dicks E, Tyrer JP, Rosenthal AN, Intermaggio MP, Fraser L, Gentry-Maharaj A, Hayward J, Philpott S, Anderson C, Edlund CK, Conti D (2015). Germline Mutations in the BRIP1, BARD1, PALB2, and NBN Genes in Women With Ovarian Cancer. J Natl Cancer Inst.

[69] Song H, Dicks E, Ramus SJ, Tyrer JP, Intermaggio MP, Hayward J, Edlund CK, Conti D, Harrington P, Fraser L, Philpott S, Anderson C, Rosenthal A (2015). Contribution of Germline Mutations in the RAD51B, RAD51C, and RAD51D Genes to Ovarian Cancer in the Population. J Clin Oncol.

[70] Pribyl LJ, Coughlin KA, Sueblinvong T, Shields K, Iizuka Y, Downs LS, Ghebre RG, Bazzaro M (2014). Method for obtaining primary ovarian cancer cells from solid specimens. J Vis Exp.

[71] Ma T, Chen Y, Zhang F, Yang CY, Wang S, Yu X (2013). RNF111-dependent neddylation activates DNA damage-induced ubiquitination. Mol Cell.

