# Supplementary material for: Increasing sensitivity to DNA damage is a potential driver for human ovarian cancer
Source: Oncotarget. 2016 Jul 6;7(31):49710–21. doi: 10.18632/oncotarget.10436 (PMC5226541; doi:10.18632/oncotarget.10436)
Supplement: Supplementary file 1 [file oncotarget-07-49710-s001.pdf]

Increasing sensitivity to DNA damage is a potential driver for human ovarian cancer

Supplementary Materials

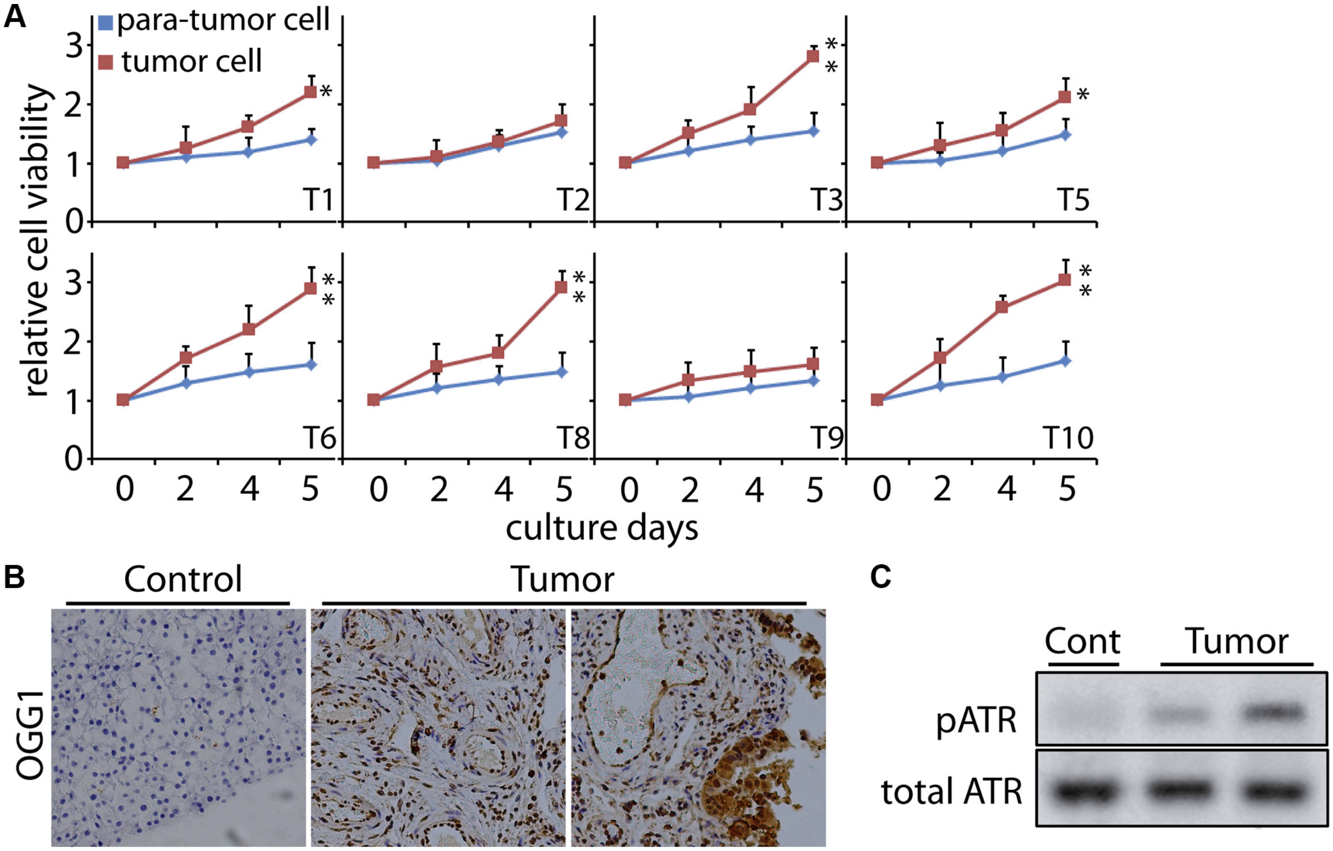

Supplementary Figure S1: Proliferation rate and DNA damage response in ovarian cancer. (**A**) The proliferation rates of para-tumor and tumor cells from different ovarian cancer samples were tested by MTT assay. * indicates the significant difference. (**B**) SSBs of para-tumor (left) and tumor (middle and right) tissues were detected by OGG1 staining. (**C**) ATR activation was detected in the para-tumor and tumor tissues. The left, middle, and right lanes are corresponding to the "left, middle, and right" in B.

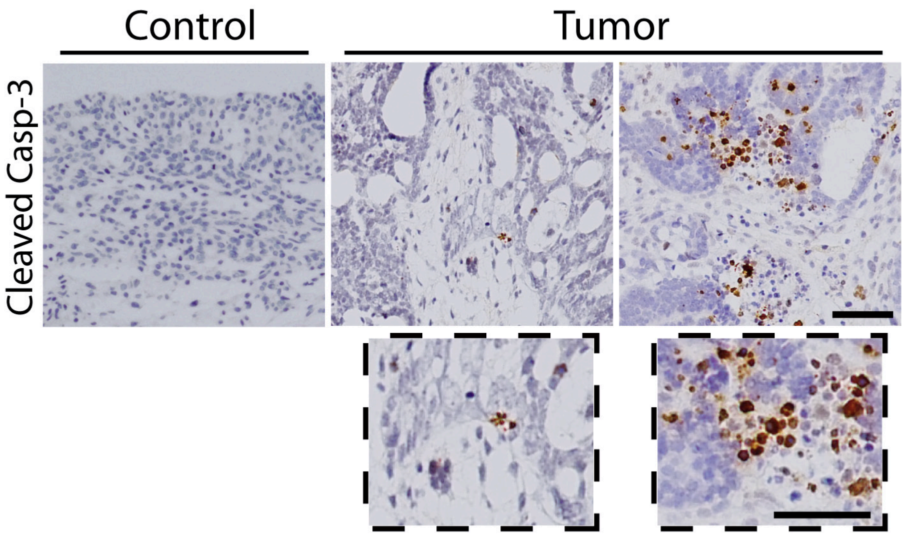

Supplementary Figure S2: Apoptosis in the ovarian cancer. Apoptotic signal was detected by cleaved caspase-3 in the para-tumor control and tumor tissues. Light (middle) and heavy (right) apoptotic signals were found among the tumor samples. The below insets show the higher magnification. Scale bars, 100 μm.

Supplementary Table S1: qPCR primers

BRCA1	Forward: TTGATGTGGAGGAGCAACAG	Reverse: CTGATTCCAGATTCCAGGTAAGG
RPA1	Forward: CAGAGAAGTTGCCAAGAGGAATA	Reverse: GGGCTGTCTAGAACCATCAAA
MRE11	Forward: CTCAGCAGCAACCAACAAAG	Reverse: GTCTGCTCTTCCTCTTTGAGAC
RNF8	Forward: GGGCTCTAATGGAAGAGCTAAA	Reverse: GCTTGCATCTTCTCCTTCTCT
RAD50	Forward: AATACGGTCTGATGCCGATG	Reverse: GCATATCCAAGGCTGTGTCT
RAD51	Forward: CGCCCTTTACAGAACAGACTAC	Reverse: ACCACTGCTACACCAAACTC
Ku70	Forward: CCAAGACCCGGACCTTTAATAC	Reverse: AGTATAATCTGACGACTCCCATAGA
Ku80	Forward: GACTTAGACATGGTGGCCATAG	Reverse: GCAGCTGCACATACACTAAAC
PARP1	Forward: GCCGAGATCATCAGGAAGTATG	Reverse: ATTCGCCTTCACGCTCTATC
53BP1	Forward: GGTTCCATCAGTCAGGTCATT	Reverse: GTTCCAACTCTTCTCCCTTCTC
PNKP	Forward: CACACTGTATTTGGTCAATGGC	Reverse: GCTCAGCATCTCTCTTCTCATC
FEN1	Forward CCGCCTGGATGATTTCTTCA	Reverse CCCAGTCTTTGCCTTCTTCTTA
OGG1	Forward TGTGTACCGAGGAGACAAGA	Reverse CCCAGTGGTGATACAGTTGAG
DDB1	Forward GGAGGTGCACAACCTACTTATC	Reverse GAAACCAGACTGAGGGCATATT
REV1	Forward CGAACAGTGACGCAGGAATA	Reverse CTTTCAGCTCCCTCTGAAGTT
NBS1	Forward GACAACAACTCCAGGACCAA	Reverse CTCTGATTCTGTGTCAGCTACG
RNF168	Forward TCTGCATGGAAATCCTCGTG	Reverse CGACAGAAGGGACAGCATAAA
Lig3	Forward CCAAATCGGAGGCTCATACA	Reverse GGGAAGGTTAGTGGCAGATT
FANCA	Forward CTTCCGAGAGGTGTTGAAAGAG	Reverse GAAGTCCTGCCGTTCAGTATC
